# Selenium Nanoparticles for Stress-Resilient Fish and Livestock

**DOI:** 10.1186/s11671-015-1073-2

**Published:** 2015-09-23

**Authors:** Biplab Sarkar, Surajit Bhattacharjee, Akshay Daware, Prosun Tribedi, K. K. Krishnani, P. S. Minhas

**Affiliations:** National Institute Abiotic Stress Management, Baramati, Pune, Maharashtra 413115 India; Department of Molecular Biology & Bioinformatics, Tripura University (A Central University), Tripura, India; Department of Microbiology, Assam Don Bosco University, Azara, Assam, India

**Keywords:** Nano-selenium, Oxidative stress, Livestock and fisheries, Biological synthesis, Growth, Immunomodulation

## Abstract

The fisheries and livestock sectors capture the highest share of protein-rich animal food and demonstrate accelerated growth as an agriculture subsidiary. Environmental pollution, climate change, as well as pathogenic invasions exert increasing stress impacts that lead the productivity momentum at a crossroads. Oxidative stress is the most common form of stress phenomenon responsible for the retardation of productivity in fisheries and livestock. Essential micronutrients play a determinant role in combating oxidative stress. Selenium, one of the essential micronutrients, appears as a potent antioxidant with reduced toxicity in its nanoscale form. In the present review, different methods of synthesis and characterization of nanoscale selenium have been discussed. The functional characterization of nano-selenium in terms of its effect on growth patterns, feed digestibility, and reproductive system has been discussed to elucidate the mechanism of action. Moreover, its anti-carcinogenic and antioxidant potentiality, antimicrobial and immunomodulatory efficacy, and fatty acid reduction in liver have been deciphered as the new phenomena of nano-selenium application. Biologically synthesized nano-selenium raises hope for pharmacologically enriched, naturally stable nanoscale selenium with high ecological viability. Hence, nano-selenium can be administered with commercial feeds for improvising stress resilience and productivity of fish and livestock.

## Review

### Introduction

The fisheries and livestock sectors represent an important agriculture subsidiary and are currently attracting wide attention because of their accelerating growth and high market demand. Among that, fishery is becoming popular as fishes are important food sources rich in simple, digestible animal proteins and beneficial lipids. Globally, fish products are indispensable to one billion individuals for protein security and particularly vital for juvenile and pregnant women [1]. Besides these, it functions as a major source of income and job creation, facilitates rural livelihood, and revamps equipment manufacturing, ice making, and processing industries. In 2010, wild fisheries and aquaculture engaged 54.8 million people globally in the primary fish productivity sector only [2]. Thus, a high growth potential has been forecasted for this sector in the coming years [3].

Currently, developing countries are passing through a phase of livestock revolution. Globally, livestock system holds $1.4 trillion asset value, provides employment to at least 1.3 billion people, and maintains the livelihood of 600 million farming communities [4]. Among total dietary value, it contributes 33 % of protein and 17 % of total kilocalorie consumption [5]. On the other hand, developed countries which showed a historically stronger potential of meat and livestock supply have almost reached its saturation in the productivity and maintained the growth momentum with a share of 53 % agricultural gross domestic product (GDP) [6]. These combinatorial high and sustained demands in developing as well as industrialized countries have created big market for professional livestock husbandry and will show heightened impetus in the near future.

Fisheries and livestock sectors are currently facing vast challenges due to high input costs (land, labor, feed, medicine, etc.), reduction in farmyard, and emergence of multiple stressors. Among different kind of stresses, abiotic stresses including climate change impact, pollution, and biotic factors like pathogenic infestation and consequent disease outcome have resulted in the depletion of productivity in fisheries and livestock [4, 7, 8]. To address such challenges, new interventions like environmental bioremediation, discovery of new drug candidates, or changes in the culture protocols are important, but supplementation and delivery of targeted enrichment including micronutrient is one of the key physiological maneuvers necessary for revamping the productivity.

Oxidative stress is a universal patho-physiological phenomenon that damages cells through continuous release of reactive oxygen species (ROS) originated largely due to the exposure to biotic and abiotic stressors [9]. The common feature of different ROS varieties is to cause damage to the biomolecule of the cell or the building blocks like DNA, protein, and lipids [10]. Cells possess an array of evolutionary conserved, non-enzymatic, and enzymatic detoxification mechanisms to prevent the effects caused by oxidative stresses. The detoxification mechanism happens to be threatened due to stress impacts which in turn enhance the extent of ROS release. Dietary administration of antioxidative supplement can be an important strategy for better production of livestock and fisheries. The potential role of selenium as a counteractive trace element for oxidative stress and inducing apoptosis in stressed cell is a good option, which has been applied successfully [11].

**Fig. 1:**
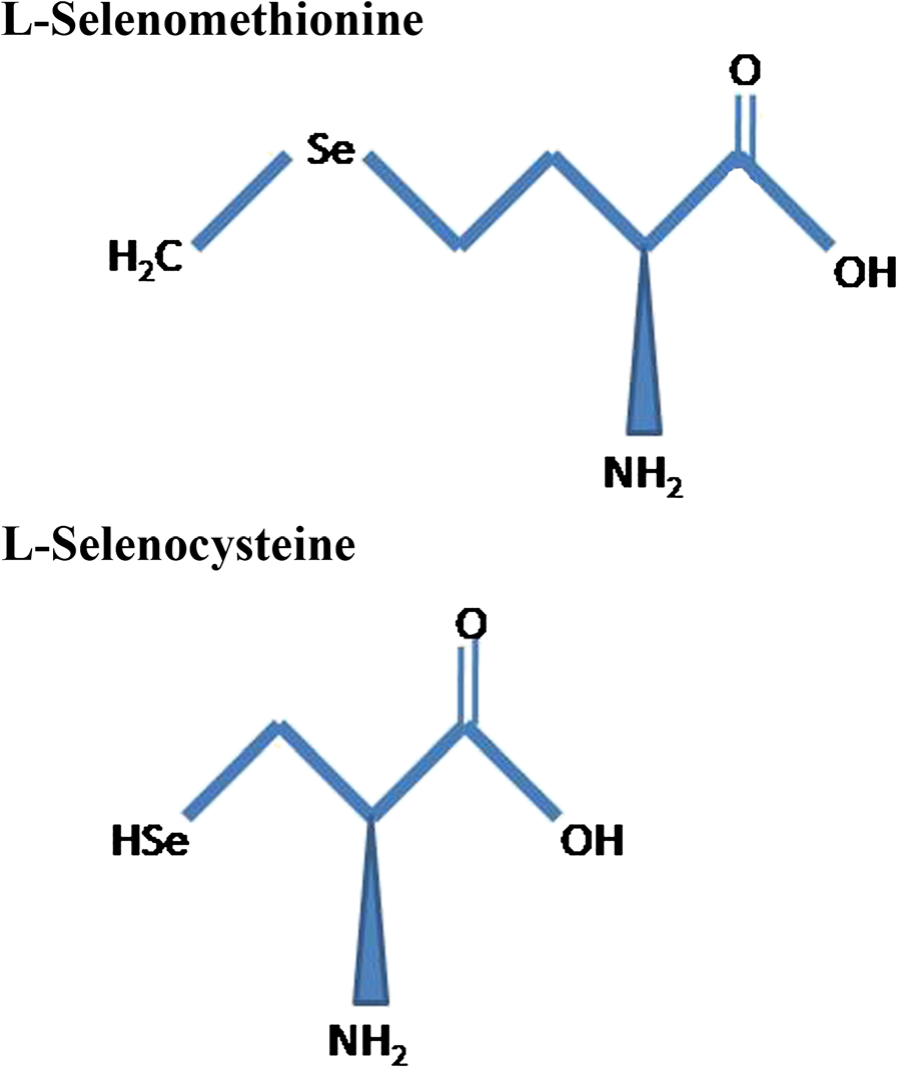
Schematic diagram depicting the structure of bioavailable selenium

The word “selenium” originates from the Greek word *Selene*, which means moon goddesses. It was discovered by Jacob Berzelius in 1818 [12]. Primarily, selenium is found immobilized in the sedimentary rocks and soils and exhibits high persistence and is closely influenced by oxidation reduction potential, pH, and solubility of soil. This immobilized selenium turns into the bioavailable form due to weathering of soil or by microbial reduction and making it available to the members of the lower tropic level of aquatic ecosystems like phytoplankton and zooplankton before its entry into higher food chain [13]. Naturally, selenium is found in both inorganic and organic forms. Selenite (Se^4+^), selenate (Se^6+^), and selenide (Se^2−^) are the three inorganic forms of selenium found in nature. Selenocysteine is a 21st amino acid, and selenomethionine is a naturally occurring selenium-conjugated amino acid that is highly bioavailable (Fig. 1) and the most suitable form of selenium for nutritional supplementation. Generally, selenium acts as a cofactor and is present in some enzymatic structures called selenoproteins in animals. The first selenoprotein identified was glutathione peroxidase (GSHPx) that helps in the catalysis of reducing hydroperoxide to respective alcohols [14]. Selenium has a major role in male reproduction, antioxidative mechanism, thyroid metabolism, anti-carcinogenesis, and muscle functioning and development [15–17]. Selenium is also an essential trace element for normal physiological function of growing animals [18]. Farm animals like livestock or poultry consume selenium primarily from plants, but all these natural selenium sources are generally low in quantity and fluctuating in their presence. To achieve time-bound, sustainable productivity in commercial farming, delivery of standard selenium dose is desirable. On the other hand, toxicity reports are also in surface as the effects of selenium administration [19]. For example, selenium treatment on the zebra fish (*Danio rerio*) embryos elicited a dose and time-dependent alteration in cardiac and neural development [20].

**Fig. 2:**
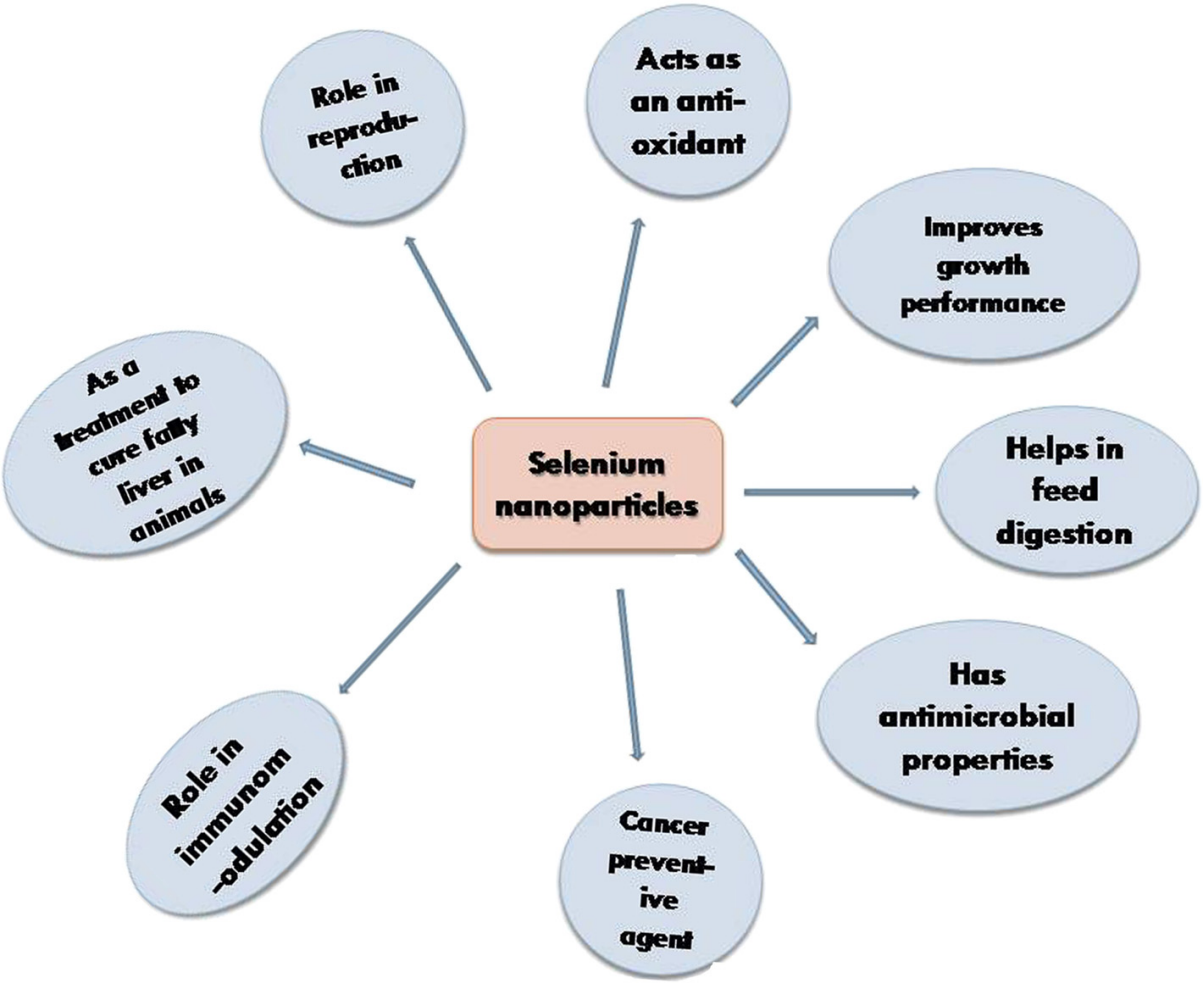
Schematic model showing different application of selenium nanoparticles

Nanotechnology is the field of science that deciphers the properties of materials at the nanoscale level. It has proved to be a great boon for modern day science and can be applied to obtain efficacious physicochemical, mechanical, and bioactive properties of various elements [21]. Among the multifaceted applications of nanoparticles in the fisheries and livestock world, reports are also appearing on enhanced efficacy of nanoscale selenium in reproduction, digestion, growth, and immunomodulation [22]. Moreover, the toxicity impact of selenium has been reduced with synthesized nano-selenium proposed by Wang et al. [23], and new physiological and biological properties such as its role in microbial inhibition and fatty liver prevention have been explored that were not elucidated earlier. Hence, nano-selenium can be administered to reduce oxidative stress and to increase the productivity of stress-ridden fish and livestock. The above mentioned functional diversity of selenium nanoparticle has been represented in a schematic diagram (Fig. 2).

### Synthesis and Characterization of Selenium Nanoparticles

#### Synthesis

Different methods have been reported to be used for the synthesis of nano-selenium including physical, chemical, and biological methods. In physical methods, hydrothermal treatments, microwave irradiation, and laser ablation are some of the important routes of synthesis for selenium nanoparticles. Quintana et al. [24] synthesized it by pulsed laser ablation method using 532-nm harmonic wavelength laser and power densities of 10^8^ W/cm^2^ with 10-ns pulse. The laser-ablated samples were deposited on silicon wafers, glass, and metallic gold films with characterization done by atomic force microscopy. The laser ablation method involves the removal of material (metal) from a solid or liquid (occasionally) surface upon irradiation with a high intensity of laser pulse energy, which converts the material into plasma. Plasma containing high concentration of metal ions is aggregated in to minute embryonic nuclei upon mutual collision, followed by slow growth and stabilization of nanoparticles [25]. Another physical synthesis was reported on t-selenium nanotubes in a hydrothermal route, following a nucleation-dissolution-recrystallization based growth mechanism. In this procedure, sodium selenite(0.5 mM), sodium hydroxide(2.4 M), and sodium formate (2 mM) were added in different ratios into an autoclave functioned at 100 °C for 25 h. A large quantity of dark-gray color-floated particles was observed on top of the solution earmarked as nanoscale selenium [26].

In chemical synthesis, different reductants have been used to synthesize nano-selenium from its precursor salt and several reports have been published regarding its chemical synthesis. It was reported that varying ratios of 100 mM sodium selenite and 50 mM ascorbic acid can be used to chemically synthesize nano-selenium and after synthesis, nanoparticle solution appeared as light orange color [27]. Ascorbic acid is a potential antioxidant and functioned in the reduction and conversion of selenium to nanoscale selenium. Dwivedi et al*.* [28] described the synthesis of spherical, 35–70-nm-sized nano-selenium using organic acid such as acetic acid and oxalic acid under ambient conditions with polyvinyl alcohol (PVA) as stabilizing agent. In its acid-induced synthesis, a carboxylic group of organic acids was found to reduce selenium salt to nanoparticles [29]. Beside acetic acid, the other carboxyl groups containing organic acids like benzoic acid and gallic acid can also be used. Similarly, selenium nanowires were reported to be synthesized by adding sodium selenite with glucose in water followed by 20-min vigorous stirring [30]. It has been hypothesized that aldehyde group of glucose is oxidized to the carboxyl group due to the nucleophilic addition of hydroxyl group (OH^−^), which eventually reduces metal ions to metal nanoparticles [31].

Recently, biological synthesis of selenium nanoparticle is gaining popularity due to its easy available source, less toxicity, and pharmacological significance [22]. Among biomaterials, synthesis was primarily reported using microbes such as bacteria and fungi with a few from plant origin. Among plants, synthesis of nano-selenium was reported in details using *Vitis vinifera* (raisin) fruit [32]. Lignin-coated selenium nanoballs of 3–18-nm size were synthesized using *V. vinifera* fruit extract and selenous acid (selenium precursor). *V. vinifera* (raisin) fruit extract was prepared by soaking shed dried fruits overnight and crushed followed by refluxing in distilled water for 30 min. Filtered extract was added to selenous acid solution and refluxed for 15 min to form spherical-shaped (nanoball) nanoparticles. Raisin constitutes flavonoids, vitamins, sugars, etc. which might be responsible for nanoparticle synthesis. Nano-selenium (20–50 nm) was prepared from *Spirulina* polysaccharides by solution phase method [33]. “Solution phase method” is an important synthetic process of combinatorial chemistry that can employ a mixture of reactants and ensure the reaction of all constituents under the conditions used. In this context, solution phase redox system was used where sodium selenite (selenium precursor) was mixed in aqueous solution of *Spirulina* polysaccharide followed by the addition of ascorbic acid solution. It was observed that ascorbic acid reduced the sodium selenite into selenium nanoparticles and *Spirulina* polysaccharide has been attached as surface decorator. These nanoparticles showed anti-cancerous activity against A375 human myeloma cell lines [33].

Among microbial sources, Fesharaki et al. [34] reported the synthesis of 245-nm-sized selenium nanoparticles from bacterial species, *Klebsiella pneumoniae*, using selenium chloride as a precursor. In this process, reduction potential of 24 h old *K. pneumoniae* culture extracts was evaluated in different culture media, from which tryptic soy broth (TSB) showed maximum reductive (1.92 mgml^−1^) potential. In another experiment, Tam et al. [35] reported the *Shewanella* sp. HN41-mediated synthesis in the presence of lactate and selenite where lactate served as an electron donor and selenite acted as a sole electron acceptor during the synthesis. The reduction of selenite is presumed to be the result of the respiratory electron transfer system and the soluble selenite reductases of the bacterial system. Singh et al. [36] reported its synthesis by *Bacillus* sp. JAPSK2 using selenium chloride as a precursor. Shrivastava et al. [37] described the biosynthesis of selenium nanostructures by bacteria *Zooglea ramigera* with selenium oxyanions. The formation of spherical crystalline monoclinic nanoparticles having a size of 30–150 nm was observed by transmission electron microscopy (TEM). It was also reported to be synthesized from selenite (sodium selenite as a precursor) stress tolerated bacteria, *Pantoea agglomerans* [38]. Intra and extracellular syntheses were reported from bacterial sp. *P. agglomerans* inoculating bacterial cells in tryptic soy broth containing 1 mM sodium selenite salt [32]. It was observed under TEM analysis that nanoparticles synthesized through intracellular route during early phase of incubation were ejected out from the cells after 24 h. The reaction mechanism involves reductases enzymes associated with cell membrane to produce selenium nanoparticles [38]. In a separate report, production of elemental nano-selenium from probiotic bacteria like *Lactobacillus casei*, *Streptococcus thermophilus*, and *Lactobacillus acidophilus* was described and it was observed that the size variability of nanoparticles depends on pH of the medium as pH plays important roles in its dissolution kinetics [39]. Some special features of biologically synthesized selenium nanoparticles have been presented in Table 1.

**Table 1:** Some of the important biological resources to synthesize selenium nanoparticles

Precursor of selenium	Organism used	Size of nanoparticles (nm)	Reference
Sodium selenite (Na_2_SeO_3_)	*Bacillus cereus* (bacteria)	150–200	[40]
Sodium selenite (Na_2_SeO_3_)	*Bacillus* sp. MSh-1 (bacteria)	80–220	[100]
Sodium selenite (Na_2_SeO_3_)	*Zooglea ramigera* (bacteria)	30–150	[37]
Sodium selenite (Na_2_SeO_3_)	*Pantoea agglomerans* (bacteria)	30–300	[38]
Selenite (Se^4+^) solution	*Klebsiella pneumonia* (bacteria)	100–550	[34]
Selenite (Se^4+^)solution	*Bacillus* sp. JAPSK2 (bacteria)	222	[36]
Sodium selenite (Na_2_SeO_3_)	*Bacillus Subtilis* (bacteria)	100	[101]
Selenium dioxide (SeO_2_) solution	*Aspergillus terreus* (fungus)	47	[102]
Selenite (Se^4+^) solution	*Shewanella sp. HN-41* (bacteria)	1–20	[35]
Sodium selenite (Na_2_SeO_3_)	*Spirulina platensis* polysaccharides (algae)	90–550	[33]
Sodium selenate (Na_2_SeO_4_)	*Alternaria alternata* (fungus)	30–150	[103]
Sodium selenite (Na_2_SeO_3_)	*Pseudomonas aerogenosa* strain JS11(bacteria)	21	[73]
Sodium hydrogen sulfate (NaHSeO_3_)	*Lactobacillus casei* (bacteria)	50–500	[39]
Sodium selenite (Na_2_SeO_3_)	*Sachharomyces cerevisiae* (yeast)	30–100	[104]

#### Characterization of Nano-selenium

The structural properties of nano-selenium is determined and characterized by studying parameters like size, shape, etc. For analyzing the structural properties, the following methods are employed: UV-visible absorption spectral analysis, X-ray diffraction analysis (XRD), Fourier transform resonance spectroscopy (FTIR) analysis, dynamic light scattering (DLS) analysis, transmission and scanning electron microscopy, etc.

UV–vis spectroscopy determines the “absorption maxima” of nanoparticles depending on the concentration of the precursor and other component of reaction mixtures. The researchers synthesized nano-selenium applying different physical, chemical, and biological methods, and among these methods, there are very less information of absorption maxima from chemical and physical routes. Though there are few reports from biological routes, these are widely varied and not confirmatory. Microbial source such as *Bacillus cereus-*mediated selenium nanoparticles showed absorption maxima at 590 nm, whereas nanoparticles synthesized from lemon leaf extract exhibited maximum absorption at 395 nm [40, 41]. Moreover, these ranges are supposed to be altered with other genotypes. Biologically synthesized nano-selenium had different absorption maxima than chemically synthesized one due to its low and variable band gap energy [42]. Band gap calculated for chemically formed nano-selenium is 2.1 eV which is significantly different from biological source (band gaps for nano-selenium from *Sulphurospirillum barnessi, Bacillus selenitireducens*, and *Selenihalanaerobacter shriftii* are 1.62, 1.67, 1.52 eV).

**Fig. 3:**
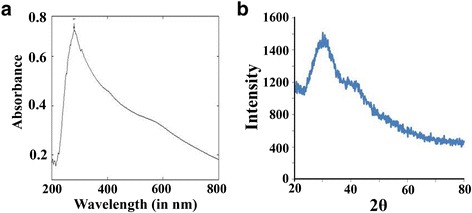
Photographic presentation of the characterized selenium nanoparticles through UV-Visible spectroscopy (**a**) and X-ray diffraction (**b**)

The absorption spectra of biologically synthesized nano-selenium from *K. pneumoniae* was demonstrated at 218 and 248 nm within 200–300-nm scan range [34]. These two absorption peaks were found because of the presence of its two different species within bacterial spheres [42]. Consistent with this information, we have synthesized selenium nanoparticles and examined its absorption spectra. The absorption spectra data showed that this nanoparticles exhibited absorption maxima at 280 nm (Fig. 3a).

X-ray diffraction technique was used to examine the composition and phase of resultant samples of nano-selenium. Nano-selenium synthesized by green method showed crystalline nature with lattice constants at *a* = 4.363 Å and *c* = 4.952 Å and XRD data are in agreement with the literature value (JCPDS File No. 06–0362) [43]. Here, “Green method” basically categorized the nanoparticles synthesis using plant, microbes, or animal origin because it is ecofriendly, benign, and less toxic and minimum additives are used. Characteristic peaks of “2θ” values for nano-selenium were observed at 23.680, 29.788, and 43.9 indicating the presence of nanoparticles synthesized from *Bacillus* sp. JAPSK2 [37]. Selenium in its nanoscale form exhibits a standard XRD pattern (23, 30, 43) which confirms its nanoscale character, and it is similar to nano-selenium originated from all different sources. In the current context, standard 2θ value confirmed its synthesis from *Bacillus* sp. JAPSK2. Consistent with these reports, we have also conducted its synthesis and examined its characteristic peaks of 2θ (Fig. 3b).

Fourier transform infrared spectroscopy (FTIR) was used to analyze the surface interaction between synthesized nanoparticles with other molecules took part in the synthesis and stabilization of nanoparticles. It was reported that nano-selenium can be synthesized by using selenite as a precursor, ascorbic acid as reducing agent, and *Spirulina* polysaccharide (SPS) as surface decorator [33]. FTIR analysis reported some weak interactions between SPS and nano-selenium (SeNPs). Analyzing FTIR spectra of SPS *(Spirulina* polysaccharide) and SPS-SeNPs (nano-selenium), a characteristic stretching vibrations of the hydroxyl group (–OH) were observed in the two spectra, but the absorption bands of –OH group at 3446 nm was shifted slightly to 3438 nm. These results suggested the presence of some weak interactions between SeNPs and SPS. The weakening of absorption intensity of –OH group indicated the decrease in free –OH group after linking SPS with nano-selenium.

Dynamic light scattering (DLS) technique was used to measure hydrodynamic effective diameters of synthesized nanoparticles. Selenium nanoparticles synthesized from dried *V. vinifera* (raisin) extract showed a zeta average diameter of 8.12 ± 2.5 nm with 0.212 polydispersity index(PDI) [32].

Scanning electron microscopy (SEM) and transmission electron microscopy (TEM) with selected area electron diffraction (SAED) are well-known techniques to determine the structure, morphology, and size of prepared nanoparticles. Nano-selenium synthesized from *Shewanella* sp. HN 41 showed the amorphous nature of nanoparticles confirmed by high-resolution TEM and SAED [35]. Another report suggested that the particle size of nanoparticles having a surface decorated with *Spirulina* polysaccharide (SPS) was in the range of 90–550 nm as determined by TEM analysis. It was further reported that the size observed was decreased with increasing concentration of SPS and reported to be 20–50 nm with homogenous spherical structure [33]. Scanning electron microscopy showed that synthesized nanoparticles exhibited spherical nanospheres with a size of 150–200 nm showing selenium-nanospheres were present freely around the cells and also present in aggregates connected to bacterial cell mass [40].

### Applications of Nano-selenium in Fish and Livestock

#### Role of Nano-selenium in Reproduction

Fish and animal breeding are the key areas of productivity enhancement, and its performance determines the future stock. Among the different causes of reproductive failure, male sterility is an important obstacle to successful breeding and fertilization where selenium has appeared as a probable solution [44]. Selenium is one of the most important elements in the male reproduction, as it is an essential component for the normal development of spermatozoa and is also incorporated into the mitochondrial capsula protein [45, 46]. Selenium (Se) is also reported to be essential for spermatogenesis, normal testicular development, and spermatozoa motility and function [47]. Among different important regulatory proteins necessary for spermatogenesis, selenoprotein P and phospholipid hydroperoxide glutathione peroxidase (PHGPx) are responsible for transporting of selenium to the testis [48, 49].

Recent studies reported that the supplementation of nano-selenium (0.3 mg/kg body weight) significantly improved the testicular selenium level, semen glutathione peroxidase activity, and ATPase activity in male boar goats as compared to control (0.06 mg/kg body weight nano-selenium supplementation). The basal level of selenium requirement is 0.5 mg/kg body weight; therefore, supplementation of 0.06 mg/kg nano-selenium in control/unsupplemented group resulted in selenium deficiency which in turn resulted in abnormal spermatozoal mitochondria and damaged spermatozoal membrane [44]. Supplementation with nano-selenium enhanced the testis selenium content and testicular and semen GSHPx activities and protected the membrane system integrity as well as the tight arrangement of the midpiece of the mitochondria [44]. Hence, nanoscale selenium appears to be more effective to enhance male reproductive efficacy than the other form of elemental selenium.

#### Nano-selenium as an Antioxidant

Biological systems and its constituent macromolecules are susceptible to oxidative damages which disrupt their structure by distorting native chemical composition. Apart from these, oxidative stress (OS) leads to the generation of reactive oxygen species (ROS) like superoxide ion, hydroxide radicals, hydrogen peroxide etc. which trigger the apoptosis in tissues. To neutralize these adverse effects of ROS, the living system uses several antioxidative defense systems including various enzymes like catalase, superoxide dismutase, glutathione-*S*-transferase and peroxidase, etc. Likewise, selenium is also an important antioxidant located at the catalytic site of thioredoxin reductase and glutathione peroxidase enzymes [50].

When nano-selenium is administered to sheep as feed supplements, it reduces the levels of thiobarbituric acid reactive substances (TBARS) in plasma indicating a decrease in lipid peroxidation [51]. Antioxidant effects of inorganic, organic, and elemental nano-selenium were studied in growing weaned Taihang black male goats in a 90-day experiment wherein higher antioxidative activities were observed in nanoparticle-treated animal compared to control [52]. An improved glutathione peroxidase activity and antioxidant status were also monitored when they were applied as dietary supplementation in a fish like crucian carp (*Carassius auratus Gibelio*) [53]. It has also been reported that nano-selenium can act as a chemopreventive agent when administered at a smaller particle size [54].

**Fig. 4:**
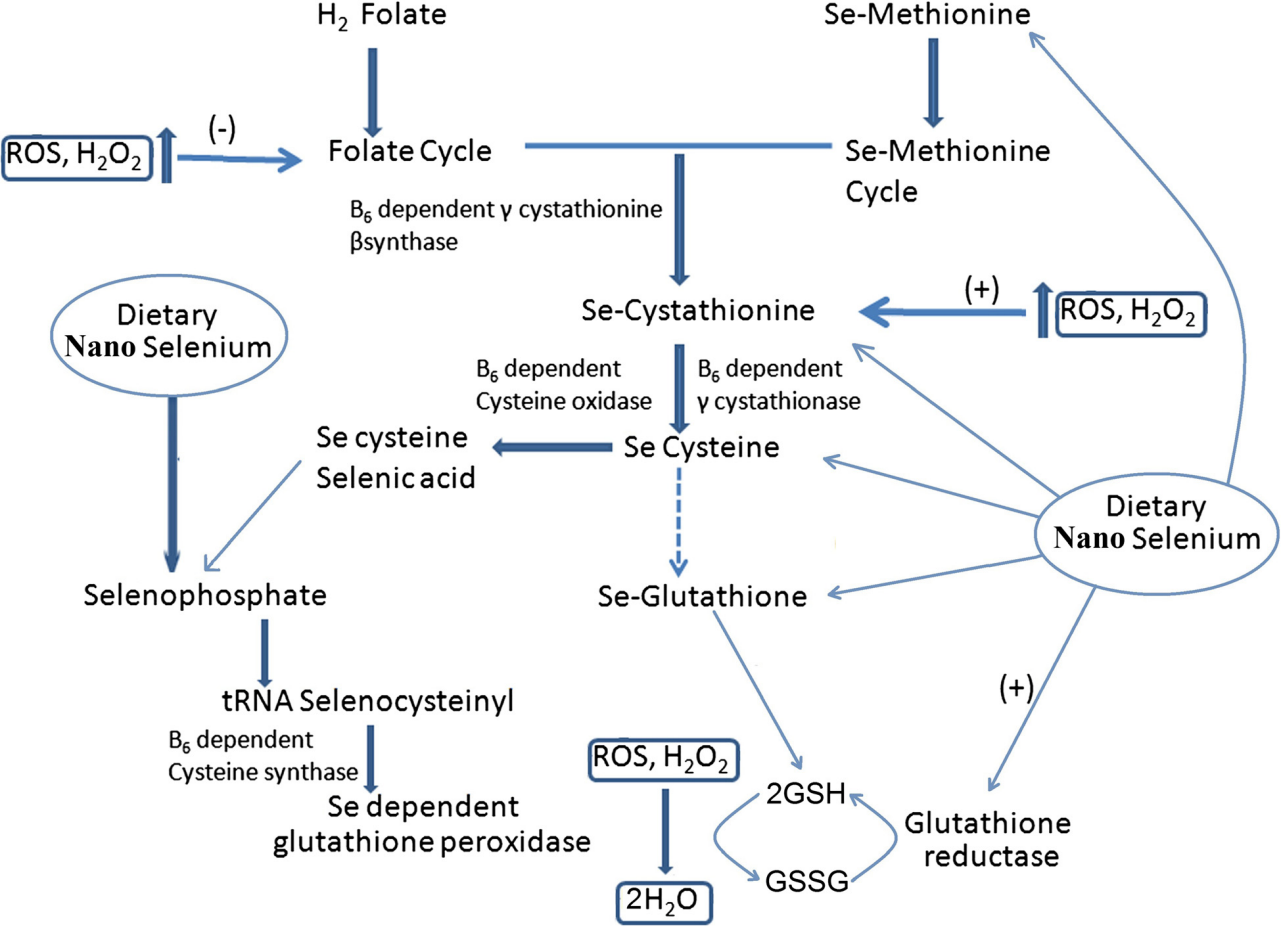
Schematic diagram representing the probable antioxidant mechanism of selenium nanoparticles

Nano-selenium showed protective effects against chromium-induced oxidative stress and cellular damage in rat thyroid [55]. It also has a potential role in correcting the levels of reduced glutathione, catalase, superoxide dismutase, and malondialdehyde due to thyrotoxicity from exposure to hexavalent chromium [55] and is also known to increase the activity of glutathione-S-transferase in comparison with selenomethionine at supra-nutritional level in mice [56]. It can be hypothesized that nano-selenium induces the formation of selenomethionine. Selenomethionine leads to the formation of the Se-cysteine from the precursor through Se-cystathionine. Se-cysteine is a constituent for the formation of Se-glutathione. Se-glutathione plays an active role in the neutralization of ROS and H_2_O_2_ in association with glutathione peroxidase. Nano-selenium reported to induce the expression of selenium-dependent glutathione peroxidase through the formation of selenophosphate, which is an integral part of tRNA selenocysteiyl [57]. Se-dependent glutathione peroxidase plays pivotal role in decreasing the ROS level inside the cell. Considering the above facts, a schematic model has been designed to showcase selenium nanoparticles induced marked attenuation in reactive oxygen species in fish and other organisms (Fig. 4).

#### Nano-selenium Improves Growth Performance

Faster growth rate through nutritional manipulation is an important facet for professional farming system. The dietary application of selenium has exhibited quick growth response in farm animals [58]. Nanoscale selenium also affects reproductive maturity and growth performance of fishes and farm animals like broiler chickens [59]. Nano-selenium when administered as feed supplement at a dose of 0.3 mg/kg to growing weaned Taihang black male goats showed an increase in body weight as compared to inorganic and organic selenium [52]. Scientists have investigated the effects of bulk selenium (Na_2_SeO_3_) and nanoscale selenium on the meat quality of finishing pigs (Duroc × Landrance × Yorkshire). It was reported that nano-selenium is effective in increasing antioxidant capacity, selenium content and decreasing drop loss in comparison with sodium selenite [60]. It exhibited higher growth performance in broiler chicken as compared to bulk selenium at concentration range of 0.4–1.0 mg/kg. Chicken fed with a dose of 0.03–1.3 mg/kg dietary selenium exhibited an increased selenium concentration in serum, breast muscle, and liver, but this increase was comparatively greater when chicken were fed with nanoparticles [61].

Nano-selenium and selenomethionine supplemented with basal diet to crucian carp (*Carassius auratus Gibelio*) improved the relative gain rate, the final weight of the fish, as well as increased the selenium concentration in muscle [53]. It was also observed that nanoscale selenium is more effective than organic selenomethionone to increase the muscle selenium content [53]. Another study with broiler chicken showed that upon nano-selenium supplementation at 2 mg/kg dose, there was a linear and a quadratic increase in selenium concentration in the muscle and liver [61]. It was also suggested that an optimal and maximum level of its supplementation could not be more than 1.0 mg/kg in broilers [62]. Plateau in survival rate, feed and gain ratio, and average daily weight gain was observed when the selenium feed concentration was 0.15–1.20 mg/kg in broiler chicken [61]. Effects of different levels of selenium supplementation on survival, growth, and reproduction of Nile tilapia were studied. It was observed that nano-selenium at a concentration of 0.6 ppm is effective to increase blood red cells counts, white blood cells, hemoglobin, and PCV% and showed best values in alanine transaminase (ALT), aspartate transaminase (AST), triglyceride, and cholesterol compared to the control diet. Therefore, the supplementation with nano-selenium at 0.6 ppm maintains the normal level of cholesterol and triglycerides in comparison to control diet in Nile tilapia [63].

#### Nano-selenium Helps in Feed Digestion

Digestion is an important physiological process in animal nutrition as it determines the assimilation and uptake of nutrients from ingested food. Xun et al. [64] reported that upon supplementation of nano-selenium and yeast selenium in male sheep (average 43.32 ± 4.8 kg body weight), the total ruminal volatile fatty acid concentration was increased, whereas ruminal pH, concentration of ammonia nitrogen(NH_3_-N), and molar proportion of propionate were decreased during the 25th day experimental regime. Selenium supplementation in sheep diet showed improved ruminal microbial activity, higher bioavailability, catalytic efficiency, and strong adsorbing ability which results into higher proficiency in digestion. Improved ruminal fermentation and feed conversion efficiency were monitored in nanoscale selenium, and it was concluded that it can be potentially used as a selenium source in ruminant nutrition [64]. It was found that the nano-selenium supplemented individual showed better tract digestibility of organic matters and its dietary supplementation improved the feed utilization.

#### Nano-selenium as Antimicrobials

Livestock including cattle and aquatic animals have been reportedly infected by pathogens leading to the decrease in their productivity, such as tilapia infected with *Streptococcus sp.* develops serious health problems or skin infection like fin rot, gill rot, etc. in rainbow trout caused by *Aeromonas bestiarum* [65, 66]. Mastitis is a most prevalent, common bacterial disease due to *S. aureus* infection in bovines triggering symptoms like decreased milk productivity [67]. Antibiotics are usually applied as a potential therapeutic agent to treat animals against major infectious diseases. But with multidrug resistance phenomenon in microbes, nanotechnology has appeared as a potential alternative for antimicrobial preparations [68]. There have been plenty of reports on antimicrobial activity of nano-formulations like silver and zinc oxide nanoparticles etc. against different pathogens where nano-selenium is a new addition [69].

Besides its role as an antioxidant and trace element in living systems, few reports have been published regarding antimicrobial potency of nano-selenium [70]. Its efficacious role on the inhibition of *Staphylococcus aureus* propagation and preventing biofilm formation has also been evaluated and reported [69, 71]. Selenium nanoparticles were also found to be effective antimicrobials against *Pseudomonas* species [36]. Coating of nano-selenium on titanium substrates has the potential to inhibit *S. epidermidis* growth as compared to uncoated materials [72]. Delivery of red elemental nanoparticles for the antibacterial study in *P. aeroginosa* strain JS-11 showed that it could serve as molecular marker for end point, qualitative, and quantitative antibacterial assays [73].

However, its antimicrobial effect should be evaluated on other multiple bacteria as well as on fungus and protozoan infections in veterinary and fishery sector. Possible mechanism of its antimicrobial function has not yet been explored, but its role on creating an osmotic imbalance or breaking some important biochemical bonds in the membrane may be predicted like other metal nanoparticles.

#### Nano-selenium and Cancer

**Fig. 5:**
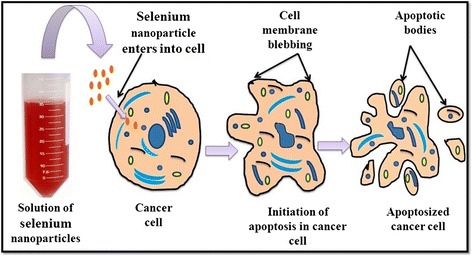
Schematic model showing the apoptosis of cancerous cell in presence of selenium nanoparticles

Cancer is a fatal animal disease referred to as a multigene disorder and is due to environmental as well as pathogenic preponderance [74]. Occurrence of cancer in domestic and farm animals has a long history and currently, rate of cancer has increased in farm animals due to environmental pollution and pathogenic outbreak [75]. Selenium is a trace element which is inserted as selenocysteine in the body via translation with the help of its own codon present on mRNA and protects the cells from oxidative damage [76]. An animal intervention study has confirmed the role of selenium as a cancer preventive agent [16]. There are reports in animals where selenium yeast reduced the risk of prostate cancer by the process of apoptosis in male dogs when supplemented with 3 or 6 μg/kg body weight per day for 7 months where no such effects were found in the control group [77]. There are several mechanisms which narrate the role of selenium as potent anti-cancer agent. Protein kinase “C” can be inactivated by the interaction of selenometabolites (CH_3_SeO_2_H) with catalytic site of protein kinase C which can lead to inhibition of tumor growth [78]. Nanoparticles in the range of 10–100-nm size are able to penetrate through cancerous tissues due to the higher pore size of blood vessels (100–800 nm) in tissues within tumors and kill them but not through healthy cells due to comparatively small pore size (2–6 nm) of blood vessels within healthy tissues [72].The anti-cancerous effect of nano-selenium on HeLa cell line was reported by Huang et al. [79], and they found that nano-selenium had potential to induce vacuolization and apoptosis in HeLa cells. They have reported that after 24-h treatment with these nanoparticles, HeLa cells exhibit vacuolization under microscope. The vacuolization and apoptotic effects of nano-selenium were found to be increased in a dose-dependent manner. Endocytosis of the nanospheres leads to membrane fusion of the vesicles which results in vacuolization [79]. An explanation for this may be the fact that vacuoles are delivered to lysosomes but cannot be degraded by acidic lysosomal enzymes, and thus, their prolonged accumulation would damage the cell leading to apoptosis [79]. A schematic model depicting the apoptosis of cancerous cell in presence of nano-selenium is represented in Fig. 5. Selenium nanoclusters coated with titanium substrates was found to inhibit cancerous osteoblast proliferation [72]. It also acts as a chemopreventive and chemotherapeutic agent for human cancer [80]. Selenium nanoparticles were found to be effective to inhibit the proliferation of human breast cancer cell line MCF-7 in a dose dependent manner [81]. Although antioxidative and anti-inflammatory properties of nano-selenium might be the basic mechanism of the anti-cancer effect, further studies are essential to elucidate the underlying mechanism of its anti-cancerous activity.

Currently, “surface decoration” is an important technique in drug delivery, and polysaccharides are attached to selenium nanoparticle to convey higher efficacy and new property. Experiments were conducted in glioblastoma where poor permeability of glioma parenchyma appears as a major limitation for anti-glioblastoma drug delivery. *Gracilaria lemaneiformis* polysaccharide (GLP) has a high binding affinity to αvβ3 integrin overexpressed in glioma cells and was employed as surface decorator to functionalize nano-selenium (SeNPs) for achieving higher anti-glioblastoma efficacy. GLP-SeNPs showed satisfactory size distribution, high stability, and selectivity between cancer and normal cells along with higher cellular uptake than its native form (SeNPs) [82].

#### Immunomodulatory Role of Nano-selenium

Oxygen is highly required for the aerobic living system, but higher oxygen concentration generates reactive oxygen species (ROS) which can cause oxidative stress [83]. Oxidative stress can be the result of either ROS over-production or decreased antioxidant defense. Since oxygen is ubiquitous for oxidative metabolism, oxidative stress response is a common phenomenon and effective defense mechanisms are required to maintain its homeostasis. Generally, antioxidative defense mechanisms are grouped as enzymatic and non-enzymatic systems. Enzymatic mechanisms of ROS detoxification are enzymatic cascades leading to complete detoxification by reacting directly with ROS or acting as redox regulators. For example, the importance of catalase could thus be seen not only for detoxification of hydrogen peroxide but consequently in adaptation to endogenous oxidative stress and lipid peroxidation also. Non-enzymatic antioxidative systems are not as specific as enzymatic ones, but nevertheless, they are in the first line of antioxidative defense and are therefore of high importance in cellular response to oxidative stress like vitamin C which quenches radicals and forms an ascorbyl radical, a stable radical which causes less oxidative damage and vitamin E which has been involved in signal transduction by modulating many specific enzyme activities and also transcription factors like NF κB [84].

**Fig. 6:**
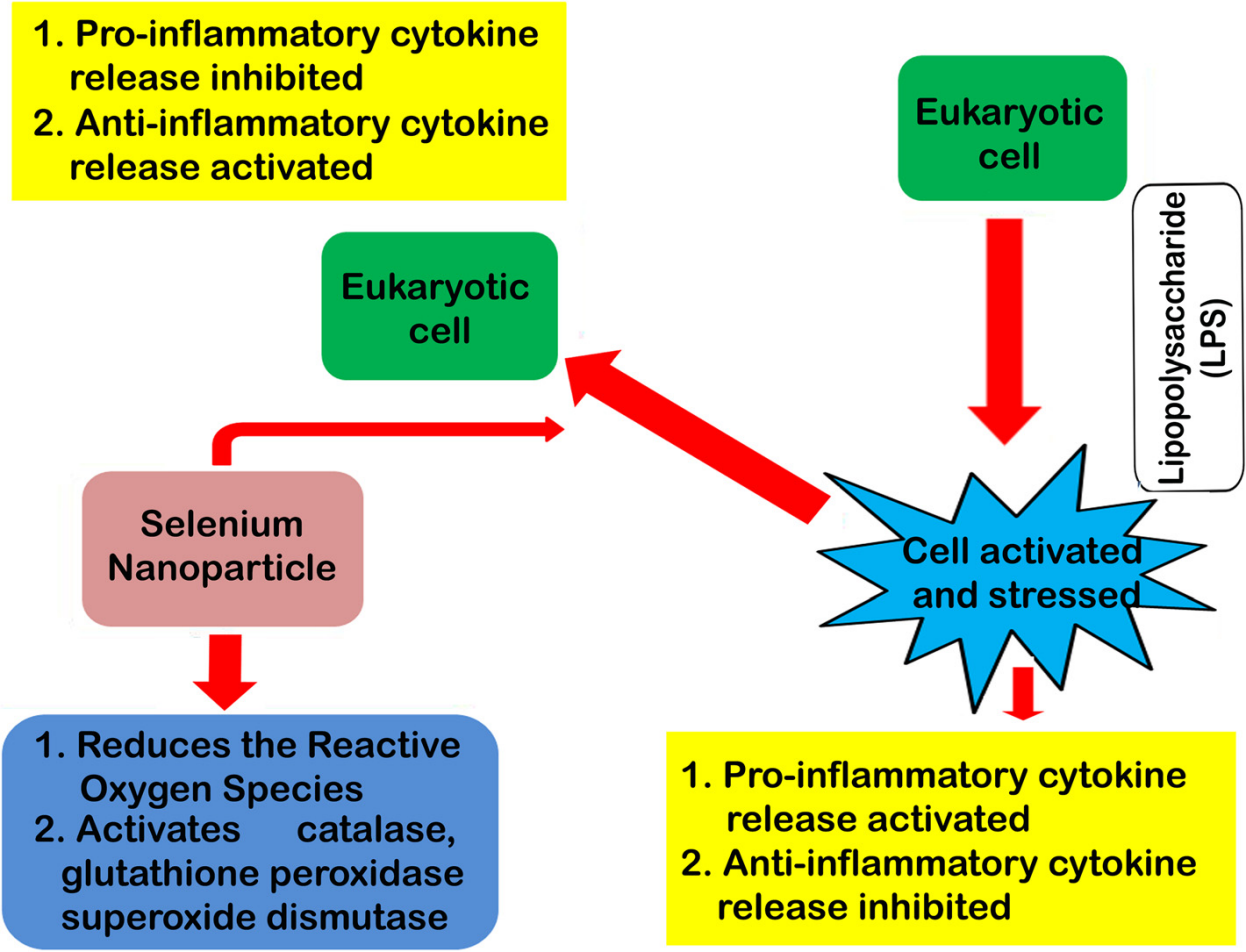
Schematic model showcasing the role of selenium nanoparticles in immunomodulation

Summarizing the knowledge of ROS and antioxidative defense mechanisms, a feedback system can be monitored in the maintenance of redox balance (oxidative homeostasis) within the cell. Efforts have also been made to synthesize multifunctional antioxidants which could be considered as “biological response modifiers” for maintaining oxidative homeostasis, both in health and in disease. Nano-selenium can be introduced for these multifunctional, versatile roles, but these important immunomodulatory phenomena are partially investigated. The role of these nanoparticles was assessed on oxidative stress induced by acetaminophen in tissues of albino rats [85]. Selenium nanoparticles decorated by sulfated *Ganoderma lucidum* polysaccharides has been shown to inhibit LPS-stimulated nitric oxide (NO) production by macrophages and down regulated mRNA gene expressions of pro-inflammatory cytokines including inducible NO synthase (iNOS), interleukin-1(IL-1), and TNF-α in a dose-dependent manner. On the other hand, the anti-inflammatory cytokine IL-10 has been markedly increased under its treatment [86, 87]. Selenium nanoparticles were also reported to increase the production of Th1 cytokines, such as IFN-γ and IL-12, in splenocytes of tumor bearing mice. The delayed type hypersensitivity (DTH) response of mice with tumor was also reported to increase in comparison to the control mice. It was reported that the survival rate of mice treated with nano-selenium was notably higher when compared to the control. From these reports, it can be suggested that its administration can result in considerable induction of the Th1 platform of the immune response through the elevation of IFN-γ and IL-12 and may be of use for the treatment of mice with tumors [88]. Considering all the above information, a schematic model has been presented in Fig. 6 showing the role of selenium nanoparticles in immunomodulation. These immunomodulatory activities can be extensively trialed in fish and livestock before its commercial application.

#### Nano-selenium Treatment to Cure Fatty Liver in Animals

Fatty liver is a sign of metabolic disorder affecting 50 % of the transition dairy cows directly after calving which is caused due to hepatic fat deposition [89]. Fatty liver can create an inflammatory response in the liver resulting a scarring and hardening and death of the cow [90]. In such cases, mortality can reach to 25 % without any proper treatment.

There are some dietary as well as anti-obesity regimes to recover from these diseases, but application of nano-selenium as feed supplement can be effective for fatty liver therapy. Role of nanoscale selenium and its supplementation were studied in male Wistar rats (200–250 g body weight) with fatty liver disease [91]. The rats were divided into four groups of eight animals each with positive and negative control and fed with and without nano-selenium diet in an experimental regime of 10 days. Redox-parameters and transmethylating ability of experimental rats were assayed along with histological examinations. In comparison to control, disease group exhibited low level inflammation and free radial release which was validated by transmethylating ability and histological analysis of the samples. This experiment demonstrated the application of bioactive nano-selenium against fatty liver disorder [91], but more animal trials are required to confirm its therapeutic details.

#### Nano-selenium Toxicity in Fishery and Livestocks

Apart from benefits of bulk and nano-form of selenium in the fisheries as well as livestock, excess dietary amount of selenium (>20–30 ppm) is toxic to most animals including livestock and fish [92, 93]. Reduction in survival rate, growth performance, feed uptake, as well as reproductive rate were monitored in fish as a consequence of selenium toxicity and have been reported to be transferred from parents to offspring [94]. Toxicity of selenium in waste water from coal fired power plant was studied over two decades on fish from Belews Lake, North Carolina. Chronic selenium toxicity was found in fish community including symptoms like telangiectasia (swelling) of gill lamellae, reduced hematocrit and hemoglobin levels, corneal cataracts, pathological alterations in major organs, reproductive failure, and teratogenic deformities [95]. Another report showed that rainbow trout (0.08 g) fry exposed to selenite at concentration 47 μg/L for 60 days resulted in reduced length and mortality [96].

Dietary concentration of selenium in excess of 3 ppm in dry feed over a longer period of exposure might become toxic to rainbow trout [95].The exact mechanism of selenium toxicity is still unclear, but there are multiple data regarding its pro-oxidant effect specially in the form of selenite, whereas selenocysteine and selenomethionine confer less toxicity [97]. Selenium at inorganic form reacts with tissue thiols like glutathione to form selenotrisulphides and also with other thiols to generate oxygen free radicals such as superoxide which trigger oxidative stress. Selenium toxicity can occur due to the formation of methyl-selenide which also creates superoxide radicals. Besides forming free radicals, selenium exhibits an inhibitory effect on thiol proteins having antioxidant effect. Hence, selenium maintains a fine balance between its antioxidant and pro-oxidant activity. The other important cause of selenium toxicity in fish and animals is bioaccumulation in tissues such as the liver, kidney, gonads, and gills at lower concentration and can be biomagnified during transfer of selenium through the food chain to higher level organism and reach to toxic levels [98].

Different sizes and doses of nano-selenium have shown variations in its impacts on cellular protein contents and enzyme activities of intracellular sodium potassium adenosine triphosphatase, lactate dehydrogenase, glutathione peroxidase, and superoxide dismutase in primary cultured intestinal epithelial cells of crucian carp, *Carassius auratus Gibelio* [21]. Evaluations on toxicity assessment of elemental nano-selenium on rats showed that nano-form of selenium is less toxic as compared to organic and inorganic forms of selenium [99]. More detailed comparative study are required in fish and animal models to elucidate the toxicity regimes, but recently developed biologically synthesized nano-selenium can be seen as a low toxicity candidate due to its ecofriendly nature containing minimum additives.

## Conclusions

Selenium is a very important trace element for counteracting stress responses in fish and livestock and showed significant efficiency at nanoscale level. Animal and fishery researchers can initiate nano-selenium work because of its more bioavailability, bioefficacy, and low toxicity. Industry can select this multipurpose feed additive against diseases like cancer, gastro enteritis, etc. or as a common antidote and immunomodulatory molecule against any single or composite stressors. As controlling oxidative stress at the first level is alternative to axing multiple diseases preponderance, application of nano-selenium can be conducted as a potential, commercial molecule to be applied to any form of delivery like drug, feed, emulsions, etc. Application of nanoscale selenium in place of precursor selenium will definitely add value to the available proximate composition of commercial “fish and animal feed” as well as to increase its selling price index. Biologically synthesized nanoscale selenium opens a wide array of options to produce ecologically viable pharmacological additives. Nano-selenium research is still in its infancy and the potentiality of this nanoparticle should be explored in order to accelerate the production in livestock and fisheries in the present stress-hit global scenario.
